# Identification and Validation of JAM-A as a Novel Prognostic and Immune Factor in Human Tumors

**DOI:** 10.3390/biomedicines12071423

**Published:** 2024-06-26

**Authors:** Tianyi Ren, You Zheng, Feichang Liu, Chenyu Liu, Bo Zhang, He Ren, Xinyue Gao, Yuexian Wei, Qiang Sun, Hongyan Huang

**Affiliations:** 1 Department of Oncology, Beijing Shijitan Hospital of Capital Medical University, Beijing 100038, China; renty@mail.ccmu.edu.cn (T.R.); lcyyyy@mail.ccmu.edu.cn (C.L.); rellian666@163.com (H.R.); 2 Frontier Biotechnology Laboratory, Beijing Institute of Biotechnology, Beijing 100071, China; zhengyousic@163.com (Y.Z.); solededlfc@163.com (F.L.); zhangbo1597@126.com (B.Z.); gxy15163855921@163.com (X.G.); 21121606@bjtu.edu.cn (Y.W.); 3 Institute of Molecular Immunology, School of Laboratory Medicine and Biotechnology, Southern Medical University, Guangzhou 510515, China; 4 College of Life Science and Bioengineering, School of Science, Beijing Jiaotong University, Beijing 100044, China

**Keywords:** JAM-A, pan-cancer, immunotherapy, prognosis

## Abstract

Junctional adhesion molecule-A (JAM-A), also known as F11 receptor (F11R), is a transmembrane glycoprotein that is involved in various biological processes, including cancer initiation and progression. However, the functional characteristics and significance of JAM-A in pan-cancer remain unexplored. In this study, we used multiple databases to gain a comprehensive understanding of JAM-A in human cancers. JAM-A was widely expressed in various tissues, mainly located on the microtubules and cell junctions. Aberrant expression of JAM-A was detected in multiple cancers at both mRNA and protein levels, which can be correlated with poorer prognosis and may be attributed to genetic alterations and down-regulated DNA methylation. JAM-A expression was also associated with immune infiltration and may affect immunotherapy responses in several cancers. Functional enrichment analysis indicated that JAM-A participated in tight junction and cancer-related pathways. In vitro experiments verified that JAM-A knockdown suppressed the proliferation and migration abilities of breast cancer cells and liver cancer cells. Overall, our study suggests that JAM-A is a pan-cancer regulator and a potential biomarker for predicting prognosis and immune-therapeutic responses for different tumors.

## 1. Introduction

Cancer is ranked as one of the leading causes of death around the world, and it is predicted to become the primary cause of premature death in this century, threatening public health and quality of life [1,2]. Traditional therapy paradigms based on tumor types have faced challenges including tumor cell plasticity, heterogeneity, and drug resistance [3,4,5,6]. Based on breakthroughs in biotechnological techniques, like massive parallel sequencing and bioinformatic improvements, the underlying molecular mechanisms of tumor occurrence, progression, and recurrence have been revealed more thoroughly and comprehensively [7,8]. With the understanding of the complexity of tumor genomics, precision medicines and individualized treatments that are gene-pointed and biomarker-based are promoted and receive more promising outcomes [9,10]. Therefore, the discovery of novel biomarkers for cancer diagnosis, progression, and prognosis is vital to improve cancer treatment methods and extend life expectancy.

JAM-A, which is also known as F11R, JAM-1, CD321, PAM-1, and JCAM, was the first member of the junctional adhesion molecule family to be discovered and is widely expressed on a variety of different cell types and enriched at interendothelial and interepithelial cell–cell junctions [11]. JAM-A is a transmembrane glycoprotein composed of an extracellular N-terminal region that contains two immunoglobulin (Ig)-like domains, the membrane-distal D1 domain and the membrane-proximal D2 domain, a single transmembrane region, and a short cytoplasmic tail on the C-terminal [12,13]. The *cis*-dimerization motif and *trans*-dimerization motif on the D1 domain participate in cell–cell contact by homophilic and heterophilic interactions [14,15]. The C-terminal PDZ domain-binding motif of JAM-A directly combines proteins in the cytoplasm to recruit signaling complexes [16]. As a key component of tight junctions (TJ) and an important adhesion molecule, JAM-A is involved in various physiological processes, including epithelial barrier function regulation [17,18], transendothelial migration of leukocytes [19,20], immune homeostasis maintenance [21,22], angiogenesis [23], central nervous system development [24], platelet aggregation, and adhesion [25].

JAM-A has been verified to participate in multiple tumor occurrence and development processes and can serve as a biomarker for several cancers [26]. JAM-A has been reported to be differentially regulated in a number of cancers including but not limited to breast cancer, lung cancer, glioblastoma, ovarian cancer, pancreatic cancer, and gastric cancer. The expression of JAM-A has also been associated with the treatment efficiency and prognosis of cancer patients [27,28,29,30]. 

Previous studies have confirmed the important role of JAM-A in tumor development and treatment; however, there is a lack of pan-cancer analysis to provide a comprehensive and all-round view of JAM-A. In this study, we used various datasets to evaluate the expression patterns, prognostic values, and functional pathways of JAM-A in pan-cancer. We also investigated potential mechanisms from genetic and epigenetic levels of the regulation. Furthermore, we investigated the correlation between JAM-A and immune infiltration as well as immune responses. Our results illustrate that JAM-A can be seen as a potential biomarker for prognosis and immunology in cancers. 

## 2. Materials and Methods

### 2.1. JAM-A Expression in Human Cancers

The expression of JAM-A in pan-cancer was analyzed using the TIMER2.0 database (http://timer.cistrome.org/), accessed on 14 January 2024, with TCGA datasets to illustrate the differential expression of JAM-A in tumor tissues and corresponding normal tissues [31]. As data for normal tissues may be inadequate in some cancers, the GEPIA2 (http://gepia2.cancer-pku.cn/) database, accessed on 25 January 2024, which combines the GTEx datasets and TCGA datasets, was employed to further confirm the differential expression of JAM-A. 

CPTAC analysis of the UALCAN portal (http://ualcan.path.uab.edu/analysis-prot.html), accessed on 25 January 2024, was utilized to explore the expression levels of JAM-A protein in multiple cancers.

The Human Protein Atlas (HPA) (http://www.proteinatlas.org/), accessed on 25 February 2024, was utilized to demonstrate the expression of JAM-A in normal and tumor tissues by immunohistochemistry staining with antibody HPA061700. Immunocytochemistry images of subcellular localization using antibody HPA043616 were also obtained from HPA.

For immunofluorescence, slides were baked at 65 °C for 1.5 h and then paraffin dewaxed using the xylene–acetaldehyde method. Antigen recovery was carried out in citrate acid buffer for 15 min after boiling using the microwave method and then blocked in 5% BSA in TBS for 1 h. Samples were first stained with JAM-A antibody (CST E6Z7E, Danvers, MA, USA) diluted 1:200 using the Opal Multiplex Tissue Staining Kit (Perkin Elmer NEL791001KT, Waltham, MA, USA) according to the standard protocol, followed by a secondary antibody using Alexa Fluor 568 anti-rabbit antibody (Invitrogen A11036, Carlsbad, CA, USA). All slides were counterstained with DAPI to show cell nuclei and then sealed with mounting medium (ZSGB-BIO, Beijing, China) and coverslips.

### 2.2. Survival Prognosis Analysis

The Cox proportional hazards regression model of overall survival (OS), disease-free interval (DFI), disease-specific survival (DSS), and progression-free interval (PFI) of JAM-A expression were determined using the TCGA datasets on the SangerBox (http://Sangerbox.com/Tool) database, accessed on 13 February 2024, using log2(X + 0.001) transformation and the logrank test for statistical testing. 

The Kaplan–Meier Plotter (http://kmplot.com/analysis/), accessed on 30 January 2024, which is sourced from databases including GEO, EGA, and TCGA was also employed to analyze the correlation between JAM-A expression and OS and disease-free survival (DFS) in different cancers using the best-performing thresholds as cutoffs [32].

### 2.3. Genetic Alteration and DNA Methylation Analysis

The cBioPortal web (https://www.cbioportal.org/), accessed on 21 February 2024, was employed for genetic mutation analysis, including the mutation frequency, mutation types, and mutation sites of JAM-A.

The UALCAN database (http://ualcan.path.uab.edu/), accessed on 16 February 2024, was used to assess the overall promoter methylation levels of JAM-A in cancers and adjacent normal tissues. Survival analysis was performed on JAM-A methylation levels using multiple probes from the MethSurv database (https://biit.cs.ut.ee/methsurv/) accessed on 17 February 2024 [33].

### 2.4. Analysis of JAM-A Expression and Immunity

We employed the “immune association” module in the TIMER 2.0 database to assess the correlation of JAM-A expression with immune infiltration analysis using TIMER, EPIC, IPS, MCPcounter, CIBERSORT, xCell, and QUANTISEQ algorithms. 

The standardized pan-cancer dataset TCGA TARGET GTEx was downloaded from UCSC (https://xenabrowser.net/) accessed on 10 January 2024. We extracted expression data from JAM-A in various samples. The ESTIMATE R package (version: 1.0.13) (https://bioinformatics.mdanderson.org/public-software/estimate/), accessed on 10 January 2024, was used to calculate the ESTIMATE scores for different types of tumors. 

The correlation of immune relative genes, tumor mutational load (TMB), and microsatellite instability (MSI) with JAM-A expression were calculated and visualized using the SangerBox database. 

### 2.5. Gene Enrichment Analysis of JAM-A

We used the STRING website (https://string-db.org/), accessed on 16 February 2024, to get the first 20 JAM-A-binding proteins and performed functional enrichment analysis of the protein–protein interaction (PPI) network. TIMER2.0 was employed to illustrate the connection between JAM-A and the binding proteins in various cancer types. 

The KEGG Pathway gene annotation was obtained from the KEGG REST API (https://www.kegg.jp/kegg/rest/keggapi.html), accessed on 25 February 2024, and the GO annotation of genes in the R software package org. Hs. eg. db (version 3.1.0) was used for gene set functional enrichment analysis. The R software package clusterProfiler (version 3.14.3) was used for enrichment analysis to obtain the results of gene set enrichment. A *p*-value of <0.05 and an FDR of <0.25 were considered statistically significant.

### 2.6. Cell Culture and Transfection 

Human liver cancer cells PLC/PRF/5 and breast cancer cells MCF7 were obtained from ATCC and cultured in MEM medium (Gibco, Grand Island, NY, USA) and DMEM medium (Gibco, USA), respectively, both containing 10% fetal bovine serum (Gibco, USA) and 1% penicillin–streptomycin (100 U/mL penicillin and 100 μg/mL streptomycin), at 37 °C in a 5% CO_2_/95% air incubator.

Cells were transfected with nontargeting control siRNAs and siRNA-JAM-As (constructed by GenePharma, Shanghai, China). Lipofectamine RNAiMAX (Invitrogen, USA) was used to introduce 10 nM siRNAs into the cells according to the manufacturer’s protocol. The corresponding phenotypic experiments were performed after 48 h of cell culture.

### 2.7. Quantitative Reverse Transcription-PCR (qRT-PCR)

The total RNA was extracted using TRIzol reagent (ThermoFisher, Waltham, MA, USA), and cDNA was reverse transcribed using the PrimeScript RT Master Mix (Takara, Tokyo, Japan). The Real-Time PCR System was used to perform qRT-PCR with the SYBR Green PCR kit (Takara, Japan). The relative expression of JAM-A was calculated using the 2^−ΔΔCt^ method and normalized with β-actin.

### 2.8. Western Blot Assay (WB)

Cells were collected and lysed in ice-cold RIPA lysis buffer (Biosharp, Hefei, China) with PMSF (Beyotime, Shanghai, China). Proteins were separated using 10% SDS-PAGE and transferred to 0.22 μm PVDF membranes, then blocked with 5% milk at room temperature for 1 h. Then, the membrane was incubated with primary antibodies anti-JAM-A (Proteintech, Wuhan, China, 1:1000) and anti-β-actin (Proteintech, 1:1000) at 4 °C overnight. After washing with Tris-buffered saline plus Tween and incubation with secondary antibodies, the Super ECL Detection Reagent (Yeasen, Shanghai, China) was used to reveal the protein blots.

### 2.9. Cell Proliferation Assay

For the cell proliferation assay, 8 × 10^4^ cells were inoculated on a 12-well plate and cultured for 72 h. The cell proliferation rate was analyzed based on the cell numbers calculated at 24 h, 48 h, and 72 h.

### 2.10. Migration Assay 

The cells were suspended in a serum-free medium and inoculated into transwell inserts at a density of 5 × 10^4^. After being cultured for 24 h, the cells that had migrated to the lower surface of the transwell inserts were immobilized with 4% PFA, stained with 0.1% crystal violet, and the relative migration rate was counted.

### 2.11. Statistical Analysis

GraphPad Prism was used for statistical analysis and presentation. Results were analyzed using a two-tailed unpaired Student’s *t*-test and presented as mean ± SD. *p* < 0.05 was considered statistically significant. The following annotations were used to indicate significance: * *p* < 0.05, ** *p* < 0.01, and *** *p* < 0.001.

## 3. Results

### 3.1. JAM-A Expression in Human Pan-Cancer

**Figure 1:**
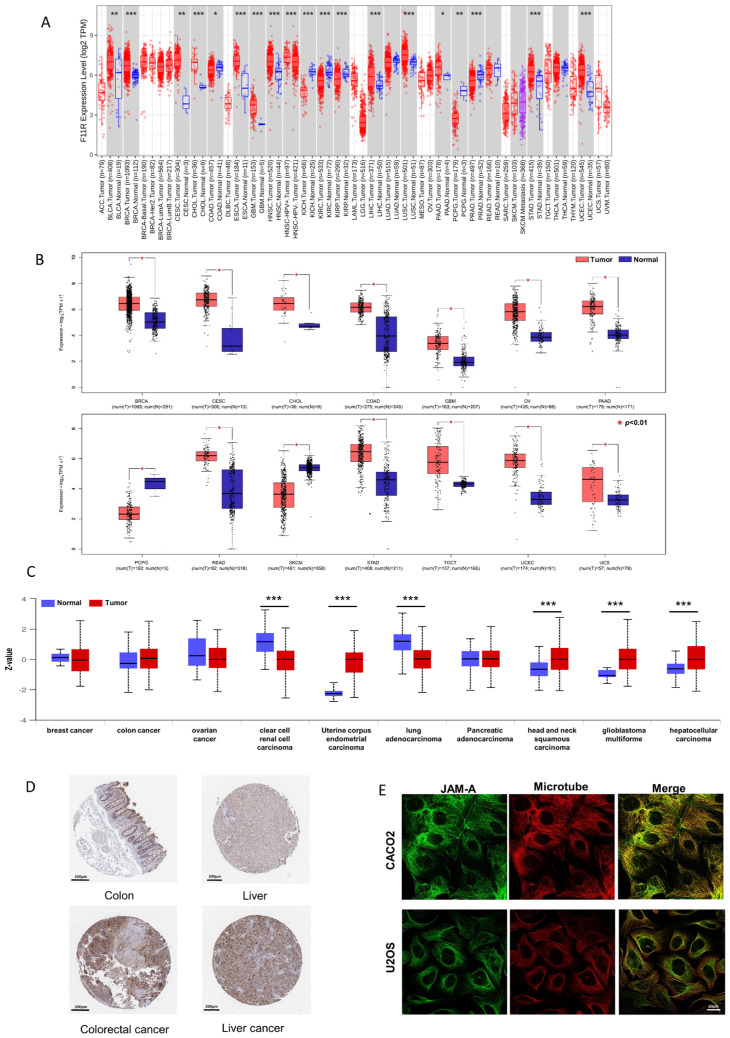
Differential expression of JAM-A/F11R. (**A**) Differential expression of JAM-A between cancer and normal samples from the TCGA dataset in TIMER2.0. (**B**) Differential expression of JAM-A between cancer and normal samples from the TCGA and GTEx datasets in GEPIA. (**C**) Total protein expression of JAM-A in cancers from CPTAC analysis. (**D**) Immunohistochemistry images of JAM-A in normal tissue and corresponding cancer tissues based on the HPA database. (**E**) Subcellular location of JAM-A in cancer cells. * *p* < 0.05, ** *p* < 0.01, *** *p* < 0.001.

To investigate the differential mRNA levels in tumor and normal tissues, we employed TCGA expression datasets from the TIMER2.0 database. As shown in Figure 1A, JAM-A was significantly up-regulated in BLCA (bladder urothelial carcinoma), BRCA (breast invasive carcinoma), CESC (cervical squamous cell carcinoma and endo-cervical adenocarcinoma), ESCA (esophageal carcinoma), GBM (glioblastoma multiforme), HNSC (head and neck squamous cell carcinoma), LIHC (liver hepatocellular carcinoma), LUSC (lung squamous cell), PAAD (Pancreatic adenocarcinoma), STAD (stomach adenocarcinoma), and UCEC (uterine corpus endometrial carcinoma). To expand the normal samples, we included the GTEx datasets in the comparison using the GEPIA2 database. Consistently higher expression was shown in BRCA, CESC, CHOL (cholangiocarcinoma), COAD (colon adenocarcinoma), GBM, OV (ovarian serous cystadenocarcinoma), PAAD, READ (rectum adenocarcinoma), STAD, TGCT (testicular germ cell tumors, UCEC, and UCS (uterine carcinosarcoma) (Figure 1B). Our immunofluorescence results in tumor and normal tissues verified the enhanced expression of JAM-A in breast cancer (Figure S1). 

We compared the differential expression levels of JAM-A protein in tumor and normal tissues using the CPTAC dataset through the UALCAN database. JAM-A protein had significantly higher expression levels in UCEC, HNSC, GBM, and LIHC (Figure 1C). We further obtained immunohistochemistry pictures of JAM-A using the HPA database. The differential expression levels between cancer tissues and corresponding normal tissues are shown in Figure 1D, in which JAM-A shows strong staining in cancer tissues. JAM-A was shown to be mainly located on the microtubes and cell junctions (Figure 1E). According to the data above, JAM-A was highly expressed in multiple cancers.

### 3.2. JAM-A Serves as a Pan-Cancer Prognostic Biomarker

**Figure 2:**
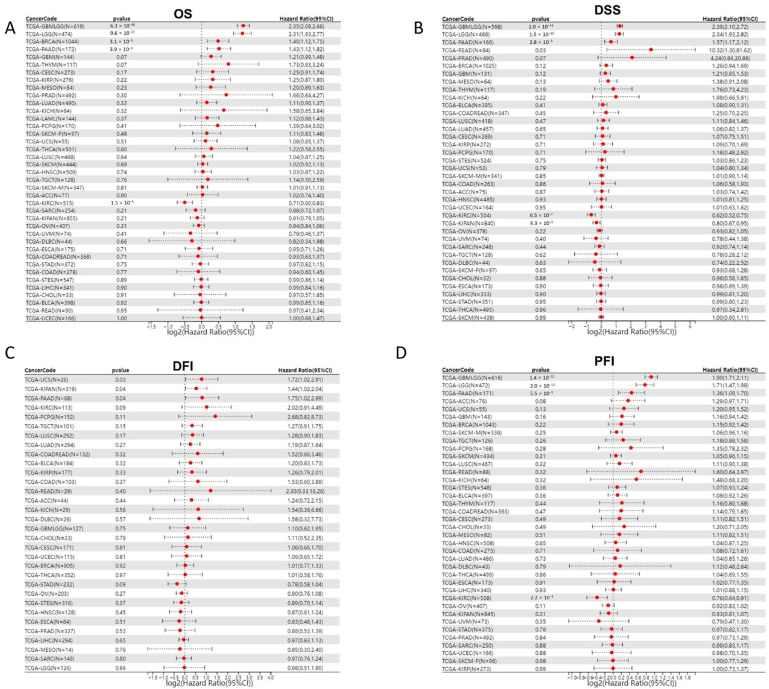
Cox proportional hazard regression of JAM-A expression and pan-cancer prognosis in the TCGA dataset. (**A**) OS, (**B**) DSS, (**C**) DFI, (**D**) PFI.

As JAM-A was over-expressed in various cancer types, we explored the association of its expression with prognosis in pan-cancer patients. We analyzed 39 cancer types from the TCGA Pan-Cancer (PANCAN, N = 10,535, G = 60,499) dataset after excluding samples with a follow-up time of fewer than 30 days and cancer species with less than 10 samples using the Cox proportional hazards regression mode. Results showed that in GBMLGG (Glioma), BRCA, and PAAD, high expression of JAM-A was significantly related to poor OS of cancer patients, whereas in KIRC, high expression of JAM-A benefited patients’ survival (Figure 2A). To evaluate the precise impact of JAM-A expression on cancer-related survival, we analyzed the DSS of cancer patients. We found that high expression levels of JAM-A in GBMLGG, LGG, READ, and PAAD were related to poorer prognosis and were related to better prognosis in KIPAN and KIRC (Figure 2B). Meanwhile, we explored the relationship of JAM-A expression with DFI. It was shown that in KIPAN, PAAD, and UCS, JAM-A was associated with shorter intervals (Figure 2C). As for the PFI, JAM-A was a high-risk gene in GBMLGG, LGG, and PAAD and a low-risk gene in KIRC (Figure 2D). 

**Figure 3:**
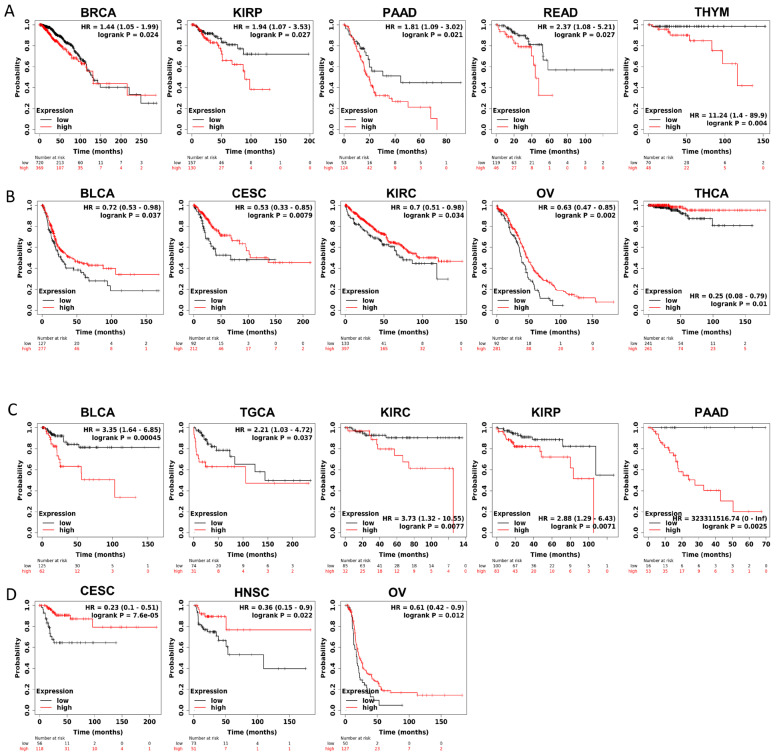
Survival prognosis analysis of cancers with JAM-A/F11R expression in Kaplan–Meier analysis. (**A**) High expression of JAM-A is negatively correlated with OS in BRCA, KIRP, PAAD, READ, and THYM. (**B**) High expression of JAM-A is positively correlated with OS in BLCA, CESC, KIRC, OV, and THCA. (**C**) High levels of JAM-A are associated with poor DFS in BLCA, TGCA, KIRC, KIRP, and PAAD. (**D**) High levels of JAM-A are associated with better DFS in CESC, HNSC, and OV.

Furthermore, to gain more details about the association of JAM-A mRNA expression with prognosis, we used the best-performing thresholds as cutoffs and displayed the Kaplan–Meier plotter of OS and DFS in each type of cancer. Figure 3A shows that high expression of JAM-A was a bad prognostic factor in BRCA, KIRP, PAAD, READ, and THYM but a protective prognostic factor in BLCA, CESC, KIRC, OV, and THCA for OS (Figure 3B). As for DFS, high expression of JAM-A was related to poorer survival in BLCA, TGCA, KIRC, KIRP, and PAAD and better survival in CESC, HNSC, and OV (Figure 3C, D).

The above results indicate that JAM-A has a significant impact on survival factors, suggesting that it plays an important role in cancer prognosis.

### 3.3. Genetic Mutation Features of JAM-A in Pan-Cancer

**Figure 4:**
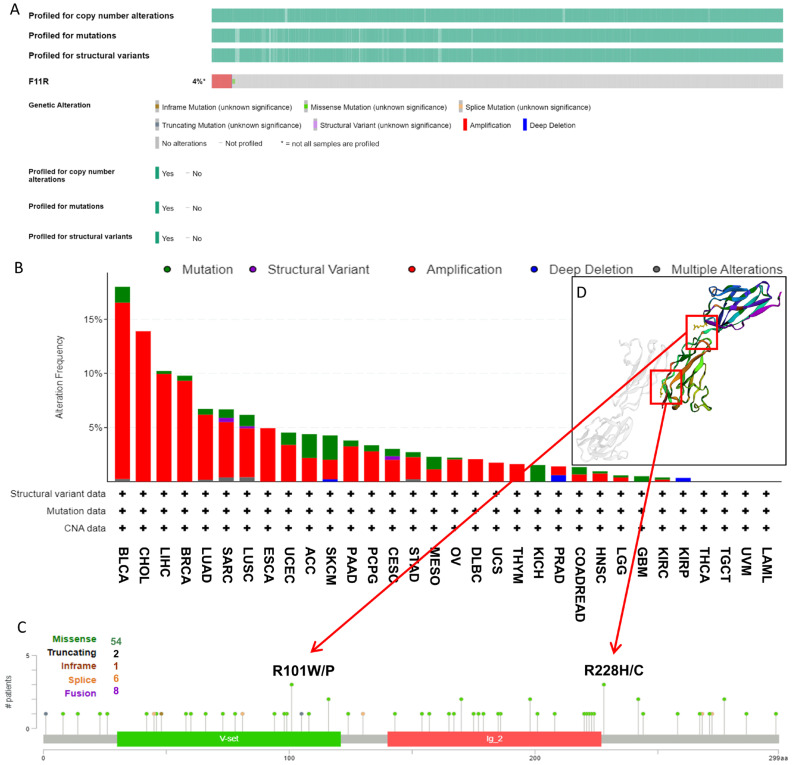
Genetic mutation features of JAM-A/F11R in pan-cancer. (**A**) The overall landscape of JAM-A in the TCGA dataset. (**B**) Mutation types and frequencies of JAM-A in different kinds of tumors. (**C**) JAM-A gene mutation sites and the number of cases. (**D**) The 3D structure of mutation sites.

As it is well known that genetic alterations are closely connected to biological function and tumor development, we analyzed the mutational profile of JAM-A in human cancers using the cBioProtal database based on TCGA datasets. As seen in Figure 4A, about 4% of patients experienced JAM-A mutations, and the leading types were amplification and missense mutation. In detail, JAM-A was altered most frequently in BLCA, with more than 15% of patients experiencing amplification or mutation, followed by CHOL with 13.89% of patients experiencing amplification. High mutation frequency also occurred in LIHC and BRCA at around 10% by amplification and mutation of JAM-A (Figure 4B). Figure 4C provides an overview of the types and sites of JAM-A genetic mutations on the amino acid sequence, in which 54 cases contain missense mutations. R101 and R228 were the most frequent sites of alteration, and the corresponding mutated sites exhibited 3D structures, as detailed in Figure 4D.

### 3.4. Epigenetic Alterations of JAM-A in Pan-Cancer

**Figure 5:**
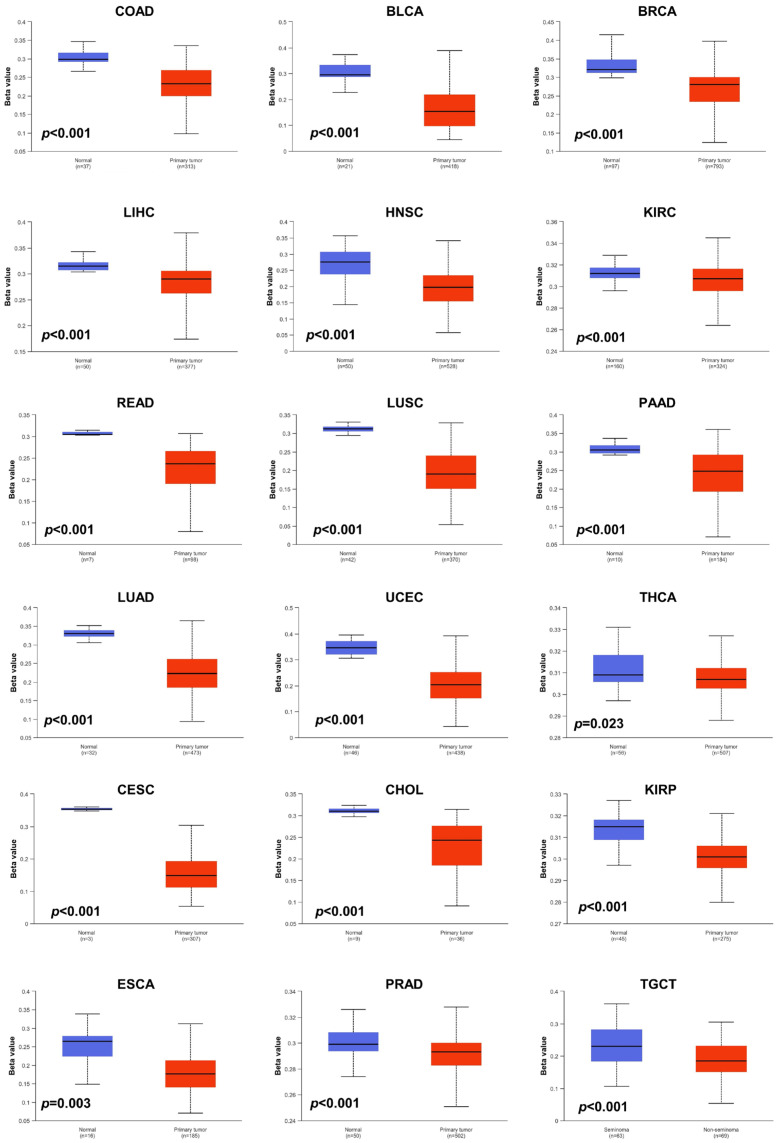
DNA methylation levels of JAM-A/F11R in pan-cancer. JAM-A was significantly hypomethylated in BLCA, BRCA, CHOL, COAD, CESC, ESCA, HNSC, KIRC, KIRP, LIHC, LUAD, LUSC, PAAD, PRAD, READ, TGCT, THCA, and UCEC.

**Figure 6:**
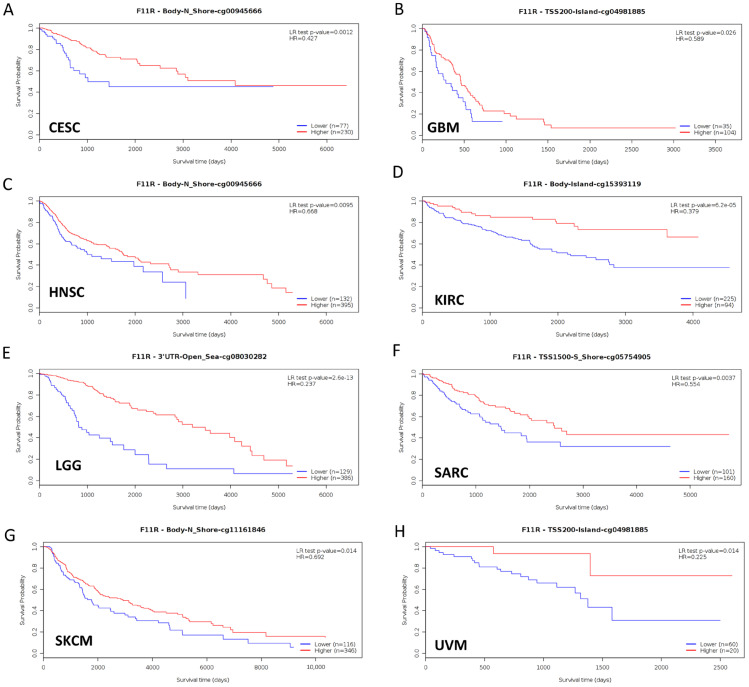
Hypomethylation of JAM-A/F11R was correlated with poor survival in CESC, GBM, HNSC, KIRC, LGG, SARC, SKCM, and UVM.

Aberrant DNA methylation levels are reported to contribute to tumor progression, so we estimated the promoter DNA methylation levels of JAM-A using the UALCAN database. The results showed that JAM-A was significantly hypomethylated in 18 types of tumors, including BLCA, BRCA, CHOL, COAD, CESC, ESCA, HNSC, KIRC, KIRP, LIHC, LUAD, LUSC, PAAD, PRAD, READ, TGCT, THCA, and UCEC (Figure 5). We also used MethSurv, which includes 14 methylation probes associated with JAM-A, to assess the relevance between the DNA methylation levels of JAM-A and prognosis. It can be seen that in CESC, GBM, HNSC, KIRC, LGG, SARC, SKCM, and UVM, most probes detected a correlation of hypomethylation with poor survival (Figure 6). These outcomes imply that JAM-A expression is affected by methylation status, which further influences tumor development and patient survival. These results suggest that epigenetic alterations regulate the expression and potential functions of JAM-A, which may have impacts on its role in different types of cancer. 

### 3.5. JAM-A Expression and Immune Infiltration

**Figure 7:**
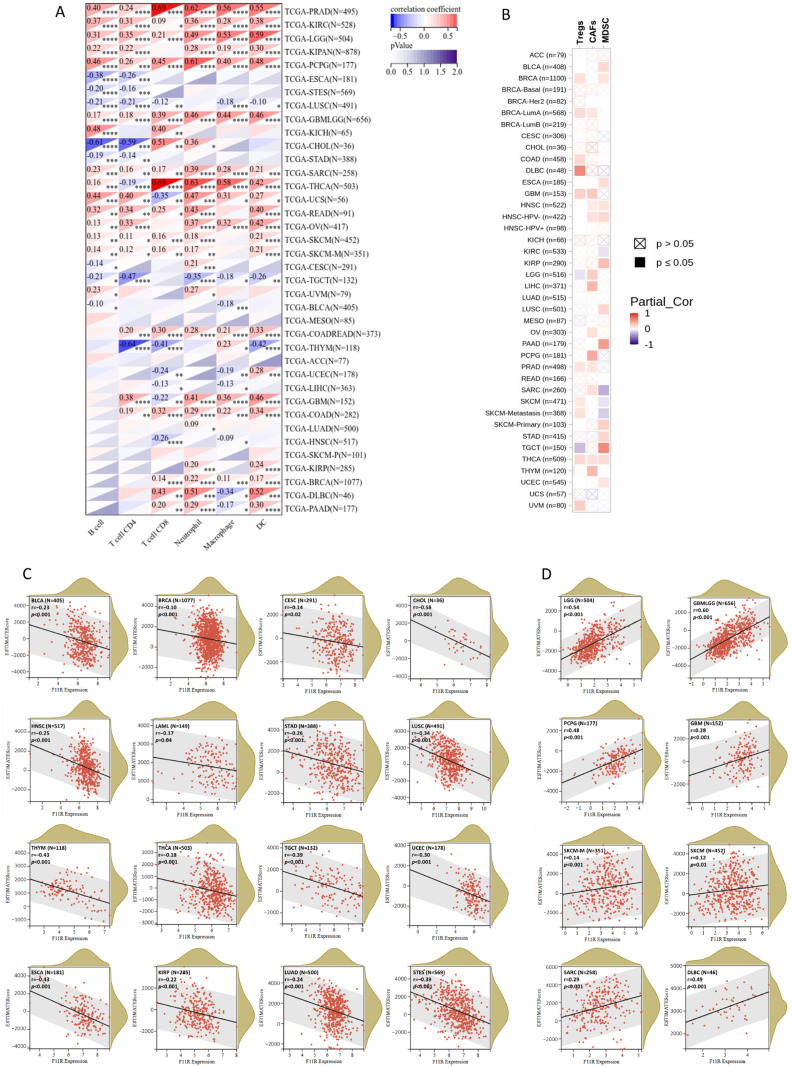
JAM-A/F11R expression and immune infiltration. (**A**,**B**) Correlation between JAM-A expression and six main immune cells (CD4 + T cells, CD8 + T cells, B cells, macrophages, DC, and neutrophils) (**A**) and three immunosuppressive cells (MDSC, CAFs, and Tregs) (**B**). (**C**,**D**) ESTIMATE score of JAM-A in pan-cancer. * *p* < 0.05, ** *p* < 0.01, *** *p* < 0.001, **** *p* < 0.0001.

Immune infiltration is a vital factor in the tumor microenvironment (TME) that can affect the occurrence and progression of tumors and plays an important role in immunotherapy and clinical efficacy. To assess the influence of JAM-A expression on the immune infiltration landscape, we explored the correlation between JAM-A expression and the infiltration levels of six main immune cells (CD4 + T cells, CD8 + T cells, B cells, macrophages, DC, and neutrophils) and three immunosuppressive cells (MDSC, CAFs, and Tregs). The outcomes demonstrated that JAM-A expression levels correlated positively with overall promotive immune infiltration in PRAD, KIRC, LGG, PCPG, GBM, and SARC and negatively in LUSC and TGCT (Figure 7A).

Meanwhile, positive associations of immunosuppressive cells were mainly observed in multiple cancer types (Figure 7B). Furthermore, we analyzed the ESTIMATE score of JAM-A in each cancer, which comprehensively calculated the purity of tumor cells and the abundance of stromal cells. The content of stromal cells and immune cells in the tumor microenvironment increased as the score increased, while the content of tumor cells decreased. As shown in Figure 7C, the ESTIMATE score was negatively connected with JAM-A expression in CESC, LUAD, LAML, BRCA, ESCA, STES, KIRP, STAD, UCEC, HNSC, LUSC, THYM, THCA, TGCT, BLCA, and CHOL and positively connected with JAM-A expression in GBM, GBMLGG, LGG, SARC, SKCM-M, SKCM, PCGC, and DLBC, as shown in Figure 7D. These results suggest that JAM-A expression is closely related to immune infiltration in pan-cancer and may have distinct impacts on different types of tumors.

### 3.6. Predictive Value of JAM-A in Evaluating Immunotherapy Response

**Figure 8:**
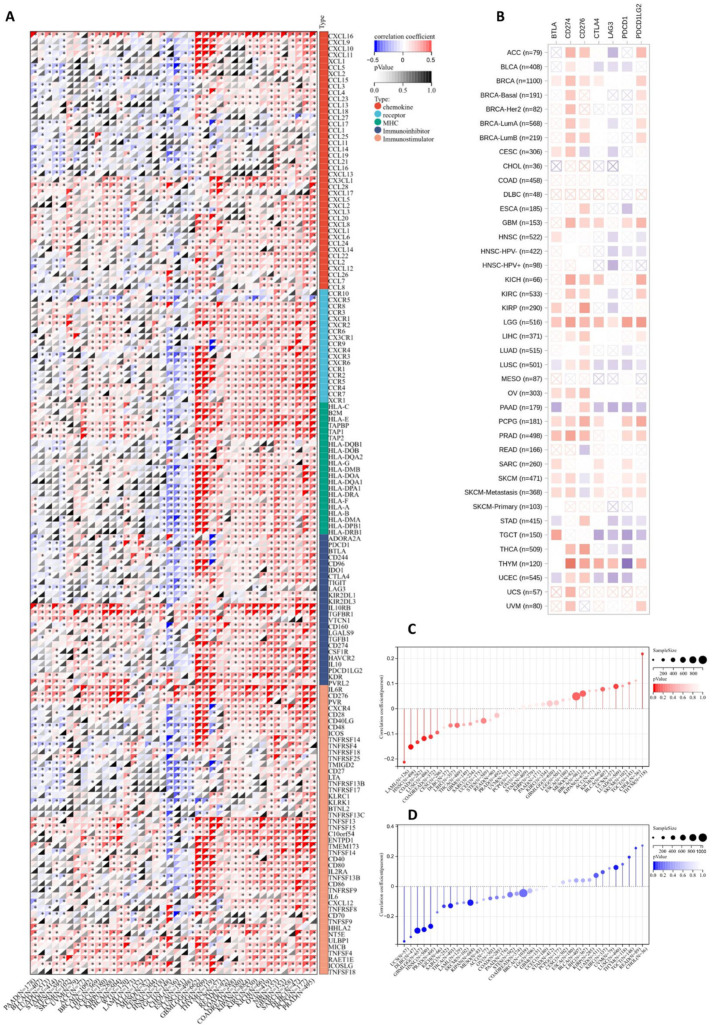
Association of JAM-A/F11R expression with immunotherapy response factors. (**A**) Analysis of JAM-A expression using 150 marker genes including chemokines, receptors, MHC, immunoinhibitors, and immunostimulators. (**B**) Analysis of JAM-A expression using the seven most common checkpoints in immunotherapy. (**C**,**D**) The relationship of JAM-A expression levels with TMB (**C**) and MSI (**D**). * *p* < 0.05.

Immunotherapy is a promising treatment method for multiple cancers, and its effectiveness is closely related to the immune microenvironment [34]. We evaluated JAM-A expression using 150 marker genes from five immune pathways, including chemokine, receptor, MHC, immunoinhibitor, and immunostimulator. The results in Figure 8A illustrate that in GBMLGG, LGG, THYM, USC, READ, COAD, COADREAD, KIPAN, KIRC, KICH, OV, NV, GBM, SARC, DLBC, PCPG, and PRAD, high expression of JAM-A is related to a positive association with most immune regulators, while in TGCT, CHOL, ESCA, and LUSC, high expression of JAM-A has the opposite impact. We extracted the seven most common checkpoints (BTLA, CD274, CD276, CTLA4, LAG3, PDCD1, and PDCD1LG2) in immunotherapy into Figure 8B, which shows that CD274 has a positive connection with high expression of JAM-A and LAG3 has a negative connection in most cancers. For LGG, highly expressed JAM-A is related to the overexpression of all seven checkpoints. 

TMB and MSI are biomarkers that can predict a patient’s response to immune checkpoint inhibitors [35,36,37]. As shown in Figure 8C, the expression levels of JAM-A were positively correlated with TMB in LUAD and THYM and negatively correlated with TMB in COAD, COADREAD, LAML, HNSC, and LUSC. As for MSI, the expression levels of JAM-A were positively connected with LUSC, READ, and TGCT, while being negatively connected in GBMLGG, SARC, KIPAN, PRAD, HNSC, THCA, UCS, and DLBC (Figure 8D). In summary, the expression of JAM-A may impact immunotherapy responses in multiple cancers, but the influence can vary for different tumor types, suggesting the complex role of JAM-A in immune regulation.

### 3.7. Enrichment Analysis of JAM-A Related Genes

**Figure 9:**
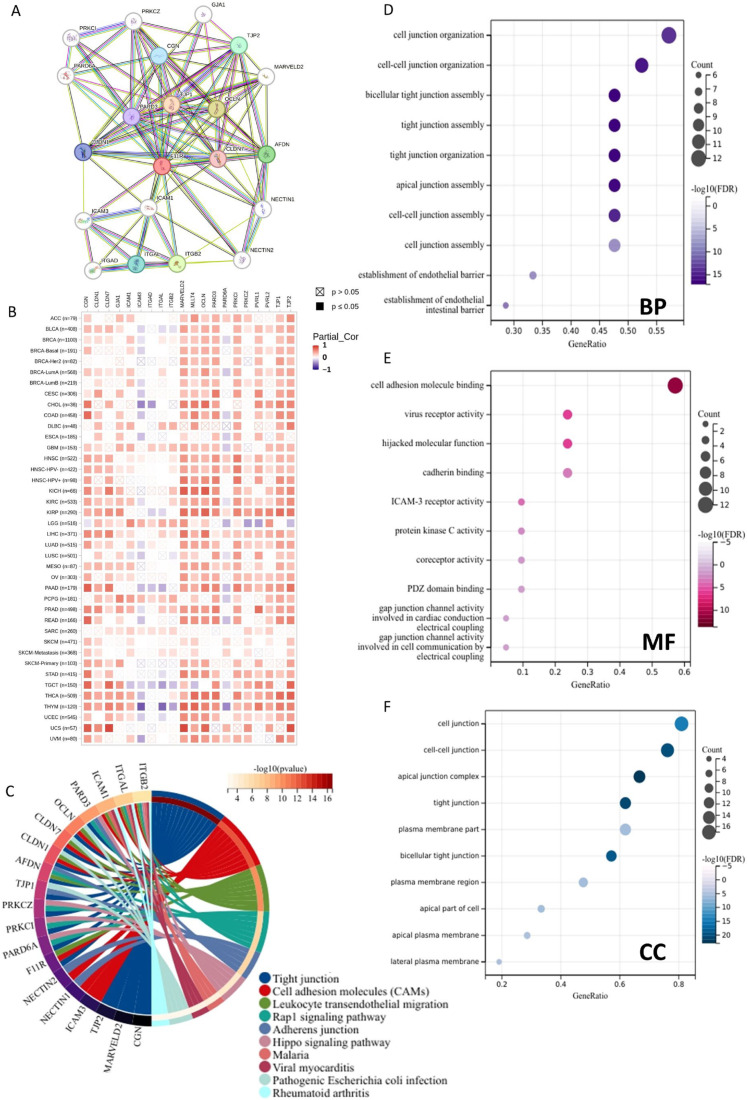
Functional enrichment analysis of JAM-A/F11R-associated genes. (**A**) Analysis of JAM-A interacting with proteins in the STRING database. (**B**) Correlation of JAM-A with 20 interacting proteins in pan-cancer. (**C**) KEGG pathway analysis of JAM-A-related genes. (**D**–**F**) GO enrichment analysis results for the JAM-A-binding proteins. (**D**) BP. (**E**) MF. (**F**) CC.

In order to gain a comprehensive understanding of the functional characteristics of JAM-A, we built a PPI network of the top 20 JAM-A-binding proteins using the STRING database (Figure 9A). We also assessed the correlation between the 20 PPI members and JAM-A in pan-cancer (Figure 9B). Furthermore, we performed functional enrichment analysis. The KEGG pathway enrichment analysis showed that the top five pathways of the proteins were tight junction, cell adhesion molecules, leukocyte transendothelial migration, rap1 signaling pathway, and adherens junction (Figure 9C). As for the GO functional annotation, the top five annotations for biological process (BP) were cell junction organization, cell–cell junction organization, bicellular tight junction assembly, tight junction assembly, tight junction organization, and cell adhesion molecule binding, virus receptor activity, hijacked molecular function, cadherin binding, ICAM-3 receptor activity for molecule function (MF), and cell junction, cell–cell junction, apical junction complex, tight junction, and the plasma membrane part of the cellular component (CC) (Figure 9D–F). These results suggest that JAM-A is involved in biological processes and related signaling pathways such as cell junctions, cell adhesion, and cell migration. These functions and pathways are deeply involved in tumor occurrence, development, and metastasis, indicating that JAM-A may play a role in tumor progression by mediating these functions and pathways.

### 3.8. JAM-A Knockdown Shows Tumor-Inhibitive Effects

**Figure 10:**
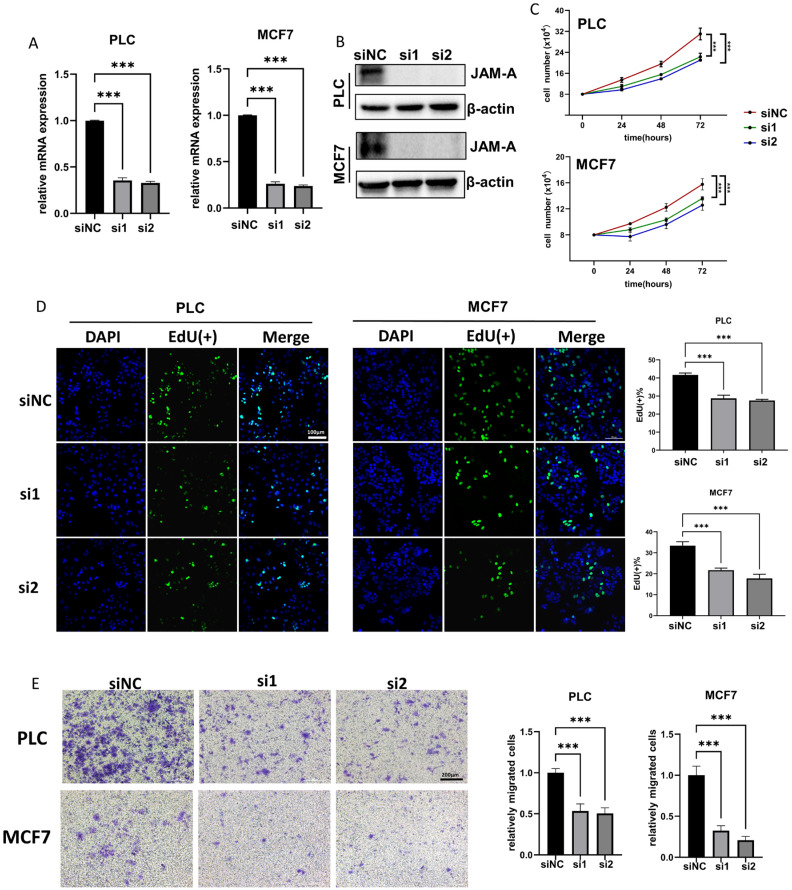
JAM-A knockdown inhibited proliferation and migration in PLC and MCF7 cells in vitro. (**A**,**B**) JAM-A knockdown verified by qRT-PCR (**A**) and WB (**B**) in PLC and MCF7 cells. (**C**, **D**) JAM-A knockdown suppressed the proliferation in PLC and MCF7 cells, as shown by proliferation assay (**C**) and EdU assay (**D**). (**E**) A transwell assay showed that the knockdown of JAM-A attenuated the migration ability of PLC and MCF7 cells. *** *p* < 0.001.

To further investigate the biological functions of JAM-A in cancer development, we used siRNA to down-regulate JAM-A expression in PLC/PRF/5 and MCF7 cells. The knockdown efficiency of JAM-A mRNA and protein was verified by qRT–PCR and WB (Figure 10A,B). Our results showed that JAM-A knockdown significantly restrained the cell proliferation (Figure 10C). It was confirmed by EdU assay that the DNA replication ability of PLC/PRF/5 and MCF7 cells was impaired after suppression of JAM-A expression (Figure 10D). We also performed a transwell assay to test the effect of JAM-A on the ability of cells to migrate. As shown in Figure 10E, markedly fewer cells migrated the transwell membrane in the JAM-A knockdown groups. Taken together, our studies demonstrate that inhibition of JAM-A expression suppresses proliferation and migration abilities in breast cancer and liver cancer cells.

## 4. Discussion

Pan-cancer analysis provides a cross-tumor landscape of gene aberration patterns and expression profiles, which benefits the integration and identification of abnormal pathways and functions and the recognition of potential biomarkers [38]. In this work, we conducted a comprehensive exploration of the expression alterations, prognostic values, and biological functions of JAM-A in pan-cancer for the first time.

We first identified that JAM-A was upregulated in multiple cancers, especially BRCA, CHOL, COAD, GBM, OV, PAAD, READ, STAD, TGCT, UCEC, and UCS, and overexpression was shown to be related to poor overall survival and a significant risk factor in BRCA, GBMLGG, and PAAD. The results were generally consistent with previous reports [29,39,40,41,42,43]. However, there is research on which low levels of JAM-A are associated with worse survival, as assessed by retrospective immunohistochemistry in pancreatic cancer [44]. In breast cancer, the expression and function of JAM-A are complicated, as there are articles showing that the invasion ability is enhanced after JAM-A reduction [45,46], while most reports are in agreement with our results that high expression of JAM-A promotes the migration and development of breast cancer cells and is related to poorer prognosis [27,47,48,49,50]. Other than the smaller sample size in the former articles, these contradictory results indicate the complex regulatory processes and diverse roles of JAM-A in tumors, implying its value for further research.

Genetic mutations can accumulate in tumor cells and alter the cellular phenotype, and some variants may even bring enhanced biofunctions and gain advantages over anti-tumor activities [51,52]. In our work, mutations of JAM-A were found in 4% of all patients, and most of them were amplification and missense mutations. More than 15% of patients with BLCA and around 10% of patients with CHOL, LIHC, and BRCA experienced JAM-A mutations. The most frequent sites of alteration were R101 and R228. Epigenetic dysregulation promotes cancer development and occurrence through disordered transcriptions [53,54]. As a vital epigenetic modification factor, aberrant methylation modifies expression and genome stability, and it is confirmed to impact tumor onset and progression [55,56,57]. Our study analyzed the methylation levels of JAM-A in cancers for the first time. The results show that hypomethylation of JAM-A can be observed in 18 types of cancers, among which low methylation levels were correlated with poor prognosis in multiple cancers including CESC, GBM, HNSC, KIRC, LGG, SARC, SKCM, and UVM. These findings imply that dysregulation of JAM-A methylation may participate in tumorigenesis, and methylation probes for JAM-A may be used as prognostic tools for pan-cancer.

The tumor microenvironment, consisting of stromal cells, immune cells, etc., plays an important role in tumor initiation, development, and metastasis. The acquisition and maintenance of cancer features, such as continuously activated growth signals, cell death resistance, angiogenesis, cell invasion, tumor-promoting inflammation initiation, and anti-tumor immune destruction, depend to varying degrees on TME. It is also considered an important factor in cancer therapy and drug resistance [34,58,59,60]. In our work, we assessed the contribution of JAM-A in immune filtration by analyzing the correlation between JAM-A expression and different types of immune cells and calculating the immune scores. We found that JAM-A was widely associated with multiple immune cells, but the association varied for different cancer types. The expression levels of JAM-A were shown to be negatively connected with the ESTIMATE scores in 16 types of cancer, which meant overexpression of JAM-A contributed to less immune infiltration and stronger tumor purity [61,62]. 

Immune checkpoint inhibitors (ICIs) have shown effective anti-tumor effects in treating various types of cancer. However, their use is limited by the inaccurate identification of patients who would benefit from the treatment. TMB and MSI have been proven to be helpful in predicting clinical response to ICI [35,37,63,64]. As seen from the results, expression of JAM-A was correlated with TMB and MSI in 12 and 10 cancer types, respectively. We also found that JAM-A was generally related to a wide range of immune marker genes in multiple cancers. These outcomes indicate that JAM-A is closely related to the immune microenvironment and has the potential to become a predictive molecule for immunotherapy.

Through the enrichment analysis of JAM-A-related genes, we discovered that JAM-A participates in tight junction, adhesion, and migration functions of cells, which were proven to be related to cancer progression [29,65]. The increase of TJ proteins may bring adhesion-independent consequences that promote cancer development [66]. We also confirmed that JAM-A knockdown suppressed the proliferation and migration of tumor cells in vitro.

There are several limitations to our work. First, validation was performed in limited tumor types, including breast cancer and liver cancer, whereas JAM-A was differentially expressed in 14 types of human tumors according to bioinformatic prediction, highlighting the need for further investigation. Second, our experimental validation primarily involved differential expression and in vitro verification of the cancer-promoting functions of JAM-A, leaving out the underlying mechanisms and specific processes through which JAM-A regulates cancer progression, such as variation and methylation patterns, immune infiltration, and other tumor-associated functions, which demand further experimentation in vivo and in vitro. Third, high levels of JAM-A expression were detected in a limited number of breast cancer samples, which, however, is far from persuasive and needs to be confirmed by examination in a larger sample size.

In summary, our study was the first one to explore the alteration and influence of JAM-A in pan-cancer. We demonstrated that JAM-A was overexpressed in most cancers, and the differential expression was correlated with methylation, immune infiltration, and immunotherapy response. These results reveal the potential of JAM-A to serve as a biomarker for pan-cancer and the value for further exploration. 

## Data Availability

Links to the datasets used in this study are given in the Materials and Methods. The results of validation experiments are shown in Section 3 part 8.

## Supplementary Materials

The following supporting information can be downloaded at: https://www.mdpi.com/article/10.3390/biomedicines12071423/s1, Figure S1. Differential expression of JAM-A in breast cancer tissues and in normal breast tissues. In tumors tissues, 6/10 showed high staining of JAM-A (A3, A4, B1, B2, B4, C1) while 2 normal tissues both showed low staining.
