# Supplementary material for: Identification and Validation of JAM-A as a Novel Prognostic and Immune Factor in Human Tumors
Source: Biomedicines. 2024 Jun 26;12(7):1423. doi: 10.3390/biomedicines12071423 (PMC11275048; doi:10.3390/biomedicines12071423)
Supplement: Supplementary file 1 [file biomedicines-12-01423-s001.zip › Figure S1.pdf]

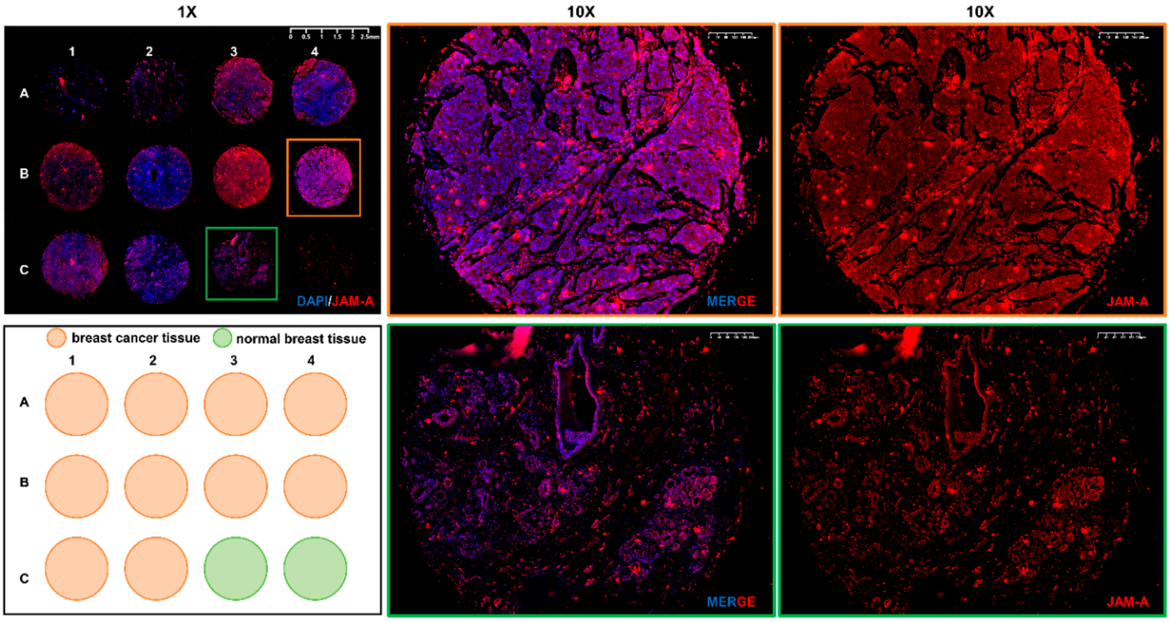

Figure S1. Differential expression of JAM-A in breast cancer tissues and in normal breast tissues. In tumors tissues, 6/10 showed high staining of JAM-A (A3, A4, B1, B2, B4, C1) while 2 normal tissues both showed low staining.
