# Supplementary figures and images for: Identification and Validation of JAM-A as a Novel Prognostic and Immune Factor in Human Tumors
Source: Biomedicines. 2024 Jun 26;12(7):1423. doi: 10.3390/biomedicines12071423 (PMC11275048; doi:10.3390/biomedicines12071423)

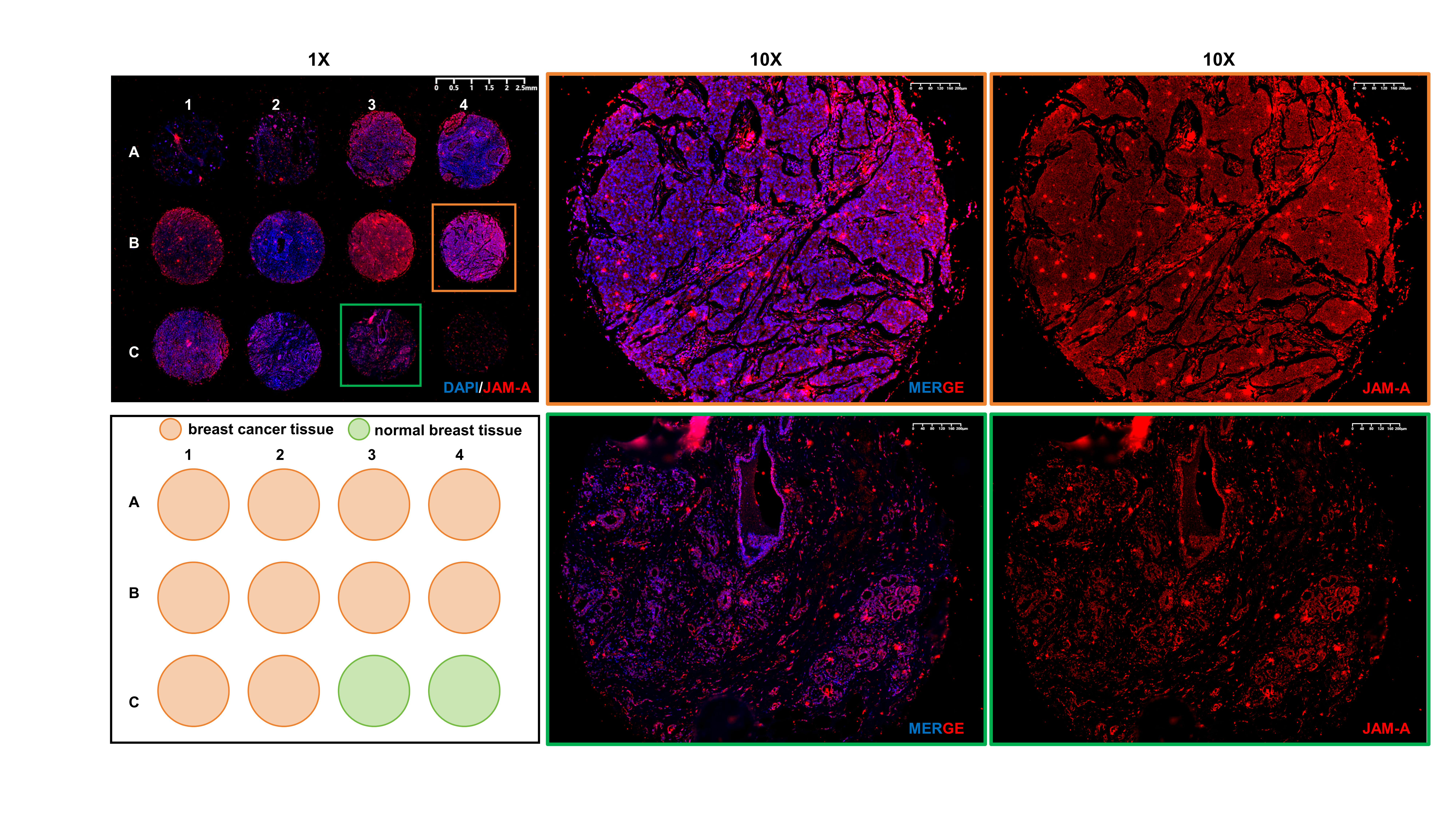

Supplement: Supplementary file 1 [file biomedicines-12-01423-s001.zip › Figure S1.tif]
